# Infectious complications and outcomes after limb salvage surgery with vascular reconstruction: A retrospective analysis from a lower middle-income country

**DOI:** 10.12669/pjms.42.(11AASC).15652

**Published:** 2026-04

**Authors:** Nadeem Ahmed Siddiqui, Javeria Saeed, Bisma Zubair, Masood Umer

**Affiliations:** 1 Nadeem Ahmed Siddiqui, FCPS Department of Surgery, Aga Khan University Hospital, Karachi, Pakistan; 2 Javeria Saeed, MSc Department of Surgery, Aga Khan University Hospital, Karachi, Pakistan; 3 Bisma Zubair, MSc Department of Surgery, Aga Khan University Hospital, Karachi, Pakistan; 4 Masood Umer, FCPS Department of Surgery, Aga Khan University Hospital, Karachi, Pakistan

**Keywords:** Functional status, Limb salvage, Sarcoma, Wound infection

## Abstract

**Objective:**

Soft tissue sarcomas can involve major blood vessels, historically leading to amputation as the primary treatment. Advances in surgical and vascular reconstruction techniques, along with multidisciplinary care, now allow for limb preservation in selected cases. However, outcomes data from resource-limited countries remain scarce. Therefore, the aim of this study was to assess the infectious, oncological, and functional outcomes of limb salvage surgery with vascular reconstruction in patients with extremity sarcoma.

**Methodology:**

A retrospective review was conducted at Aga Khan University Hospital, Karachi, Pakistan including 29 patients (12 females, 17 males; mean age 34.6 years) who underwent elective sarcoma excision with vascular intervention from 2002 to 2024. All patients had preoperative metastatic screening and MRI to assess tumor extent and vascular involvement. Vascular reconstruction was performed using autologous or synthetic grafts, or primary repair, as appropriate. Outcomes assessed included graft patency, limb perfusion, functional status using Toronto Extremity Salvage Score (TESS), infection and other complications, recurrence and survival

**Results:**

Of the 29 patients, 89.6% had lower limb tumors, predominantly soft tissue sarcomas. Vascular reconstruction was required in 69% of cases. Postoperative arterial patency was 93.1%, and limb salvage was achieved in 82.7%. The mean Toronto Extremity Salvage Score (TESS) was 84.2, with 79% of patients achieving good to excellent function. Complications included wound infections (20.6%), graft infections (6.9%), and thrombosis (6.9%). Local recurrence occurred in 13.8% and distant metastasis in 24.1%. The mean follow-up was 55.2 months, with a 20.7% mortality rate due to metastatic disease. Overall mean survival was 8.6 years while five years survival rate was 73%.

**Conclusion:**

Limb salvage with vascular reconstruction for extremity sarcomas is feasible and effective, offering good functional outcomes. Although postoperative complications may occur but they are manageable with amputation reserved as a last option.

## INTRODUCTION

Soft tissue sarcomas represent a diverse category of malignancies originating from mesenchymal tissues, with the potential to affect surrounding structures, including major vascular components. Achieving clear surgical margins often requires extensive resection of these tumors, which can be complicated by the involvement of surrounding blood vessels.^1^ In earlier practices, involvement of major blood vessels was regarded as a limitation for limb salvage surgeries, resulting in amputations as the primary treatment option.^2^ However, over previous 30 years, advancements in surgical techniques and a multidisciplinary approach have transformed the era of sarcoma surgery. Vascular reconstruction is now possible to preserve limbs while maintaining functional and oncological outcomes.^3^,^4^ Amputation is considered when there is a vast chance of functional impairment of limb after wide margin excision. It is done when there is an involvement of major nerves playing role in limb functioning, infiltrative nature of lesion, skip lesions or regional or distant metastasis.^5^

Major anatomical features may need to be sacrificed to prevent the tumor from rupturing during surgery. The bone may be excised if there is any bony involvement of tumor, which can be reconstructed by bone grafts or tumor prosthesis. The sacrificed blood vessels can effectively be reconstructed by using autologous or synthetic vascular grafts while the remaining surgical defects can be covered by the help of various types of flaps and skin grafts.^3^

Arterial reconstruction after soft tissue resection is necessary to spare functionality and perfusion of limb while venous reconstruction is required to prevent limb edema and venous congestion. However, the necessity of venous reconstruction remains a subject of debate. Some surgeons prefer venous reconstruction as mandatory while others did not find any significant improvement of limb edema in arterial reconstruction vs combined arterial and venous reconstruction.^6^,^7^ Vascular grafts such as greater saphenous vein graft or alloplastic graft such as PTFE (polytetrafluoroethylene or Dacron) can be used as interposition graft conduits.^8^

Limb salvage surgery with vascular reconstruction has emerged as a vital option for achieving oncologic control while preserving function. However, these complex procedures are often associated with complications. Such post-operative complications can delay the initiation of adjuvant therapy and may adversely influence oncologic outcomes.^9^ Despite advancements in surgical techniques, the morbidities associated with limb salvage involving vascular reconstruction remain underreported, particularly in lower middle-income countries where postoperative care resources are limited. Postoperative issues such as limb edema, wound related complications or graft related complications can further compromise recovery.^10^ In this context, understanding both infectious and functional outcomes are essential for improving patient care and long-term survival.

In our part of the world only a few institutions have the capacity to facilitate these complicated procedures. Hence, as per our knowledge, the outcomes particularly regarding postoperative infection, post-operative functional status, graft patency, tumor free interval and time to recurrence of such procedures have not been reported Therefore, the aim of this study was to assess the infectious, oncological, and functional outcomes of limb salvage surgery with vascular reconstruction in patients with extremity sarcoma treated at a tertiary care center in a lower middle-income country.

## METHODS

This is a retrospective case review conducted at the Aga Khan University Hospital, Karachi using a multidisciplinary team approach for treatment of extremity sarcomas. This team consisted of a subspecialty trained musculoskeletal tumor surgeon, a vascular surgeon and a dedicated team of musculoskeletal oncologists.

### Ethical approval:

Approval from the Ethics Review Committee of Aga Khan University Hospital was obtained before the start of the study. Ethics committee exemption was taken on June 20, 2017 for including data from tumor registry from 2010 onwards retrospectively. Approval number is 4760-SUR-erc-17.

### Inclusion criteria:

- Patients who electively underwent excision of extremity sarcomas with vascular reconstruction/repair for curative and limb preserving intent from January 2010 to December 2024 were included in the study.

### Exclusion criteria:

- Patients with missing data or incomplete records were excluded.

### Patient details:

All patients underwent pre-operative metastatic screening which included CT chest and bone scan. MRI with contrast of the affected extremity was done as per routine to check for the extent of the sarcoma and invasion into adjacent neuro-vascular structures. The vascular involvement was characterized into 4 types as described by Schwartbach et al. Type-I: artery and vein, Type-II: Artery, Type-III: vein and Type-IV: none (only abutment) 11. All patients with vascular involvement were evaluated preoperatively by vascular surgeon to assess resectability and to plan reconstruction.

The primary outcomes of the study were assessment of infectious complications (superficial surgical site infection, deep wound infection, graft infection and thrombosis) and post op morbidity. The secondary outcomes included evaluation of graft patency, limb perfusion, overall survival and recurrence.

Patients were seen in orthopedics, vascular and oncology clinic at two weeks, four weeks, six months and yearly thereafter for routine surveillance. Clinical examination, US doppler, MRI and in some cases CT angiography were done to assess recurrence and graft patency. The Toronto Extremity Salvage Score (TESS) questionnaire was administered to patients via direct or telephonic interviews. All interviews were conducted by a trained clinical research coordinator who was not involved in the patients’ clinical care, to minimize interviewer bias and ensure consistency in data collection. The final score was calculated using the formula described by Devis et al.^12^ Survival was confirmed by either clinic visits or verbal communication with patient or attendant via telephonic communication.

### Statistical plan:

SPSS version 23 was used for statistical analysis. Frequency tables were for demographic, clinical and other desired characteristics. Categorical data was presented as frequencies and percentages. Continuous data as mean +/- standard deviation. Kaplan-Meier estimations were used to analyze overall survival.

## RESULTS

There was a total of 29 patients in our study, out of whom 12 were females and 17 were males. The mean age of our patients was 34.6 ± 15.05 years (17-61 years). All the patients were either ASA level 1 (with no comorbidities) or ASA level 2 with minor and controlled comorbidities such as diabetes mellitus and hypertension. The sample predominantly consisted of soft tissue tumors followed by bone tumors. Soft tissues included synovial sarcoma, liposarcoma, epithelioid sarcoma, pigmented villonodular synovitis, desmoid fibromatosis while the bone sarcoma included mainly osteosarcoma, Ewing’s sarcoma etc. The details of histopathological types of tumors, demographics of patients and type of vascular involvement can be observed from Table-I. Vascular reconstruction procedure was done in 20 patients while nine patients had primary repair of the vessel. The details of reconstruction/repair and types of grafts used for reconstruction can be found in Table-I.

**Table-I:** Patient demographics, tumor characteristics, type of involvement, methods and type of reconstruction (n = 29).

Variable	Value
Total patients	29
Gender	12 females (41.4%), 17 males (58.6%)
Mean age	34.6 ± 15.05 years (range: 17–61)
Tumor location	Lower limb: 26 (89.6%)
	Upper limb: 3 (10.3%)
Tumor type	Soft tissue: 20 (70%)
	Bone: 9 (30%)
Histopathological type	Atypical lipomatous: 1
	Chondrosarcoma: 1
	Desmoid fibromatosis: 2
	Diffuse large b-cell: 1
	Epithelioid sarcoma: 1
	Ewing’s sarcoma: 1
	Fetal rhabdomyoma: 2
	Glomus tumor: 1
	Leiomyosarcoma: 1
	Myxofibrosarcoma: 1
	Myxoid liposarcoma: 3
	Osteosarcoma: 6
	Pleomorphic sarcoma: 1
	Pigmented villonodular synovitis: 1
	Recurrent giant cell: 1
	Synovial sarcoma: 5
Vascular involvement	Type I (Artery + vein): 10 (34.5%)
	Type II (Arterial only): 15 (51.7%)
	Type III (Venous only): 2 (6.9%)
	Type IV (Abutment only): 2 (6.9%)
Procedure performed	Vascular reconstruction: 20 (69%)
	Primary repair: 9 (31%)
Type of graft used (n=20 reconstructions)	Synthetic (Polytetrafluoroethylene) graft: 7
	Reverse saphenous vein graft: 10
	Superficial femoral vein graft: 3

The mean Toronto Extremity Salvage Score (TESS) was calculated to be 84.2 ± 15.7 with a minimum score of 38 and the maximum being 98. According to the grading system used by Devis (10), only 21% had a poor outcome while 79% had good to excellent results.

The mean operative time was 253.7 ± 120.6 mins and the mean blood loss during surgery was 635.36 ml. Post operatively pulses were absent in two patients. Intraoperative complications included iatrogenic damage to ureter while post-operative complications included superficial surgical site infections, deep wound infection, graft infection and graft thrombosis. There was no statistically significant difference (p > 0.05) in wound infection rates between the autogenous graft group, synthetic graft group, and primary vascular repair group. The frequency of complications can be seen in Table-II.

**Table-II:** Surgical Outcomes, Complications, recurrence and Follow-Up.

Outcomes	Value
Graft patency (palpable pulses post-op)	27 patients (93.1%)
Absent pulses post-op	2 patients (1 PTFE, 1 SFV graft)
Mean Toronto Extremity Salvage Score	84.2 ± 15.7 (range: 38–98)
Intraoperative complications	Iatrogenic ureteral injury: 1
Post-operative complications	Superficial surgical site infection: 4
	Deep surgical site infection: 2
	Graft infection: 2
	Graft thrombosis: 2
Local recurrence only	4 patients (13.8%)
Distant metastasis only	7 patients (24.1%)
Both local recurrence + distant metastasis	1 patient
Mean tumor-free interval (local recurrence)	40.5 ± 51.41 months (range: 6–117)
Mean tumor-free interval (distant metastasis)	29 ± 27.86 months (range: 2–86)
Amputations	5 patients (17.2%)
Mortality (till last follow-up)	6 patients (20.7%)
Mean follow-up duration	55.2 months (range: 12–132)

Local recurrence of disease was also observed along with distant metastasis. Patients who had primary repair done, five out nine of them developed recurrence, four out of ten patients who underwent reverse saphenous vein graft developed recurrence, two out of three patients who underwent superficial femoral vein graft reconstruction developed recurrence while two out of seven of the patients that had PTFE graft developed recurrence. The limb salvage was achieved in 82.7% patients (n=24) and due to extensive deep wound infection, graft thrombosis with dry gangrene and graft infection so they ended up with amputation. Till the last follow-up, six (20.7%) patients in our study have expired and all six expiries were due to metastatic disease, Table-II.

**Fig.1:**
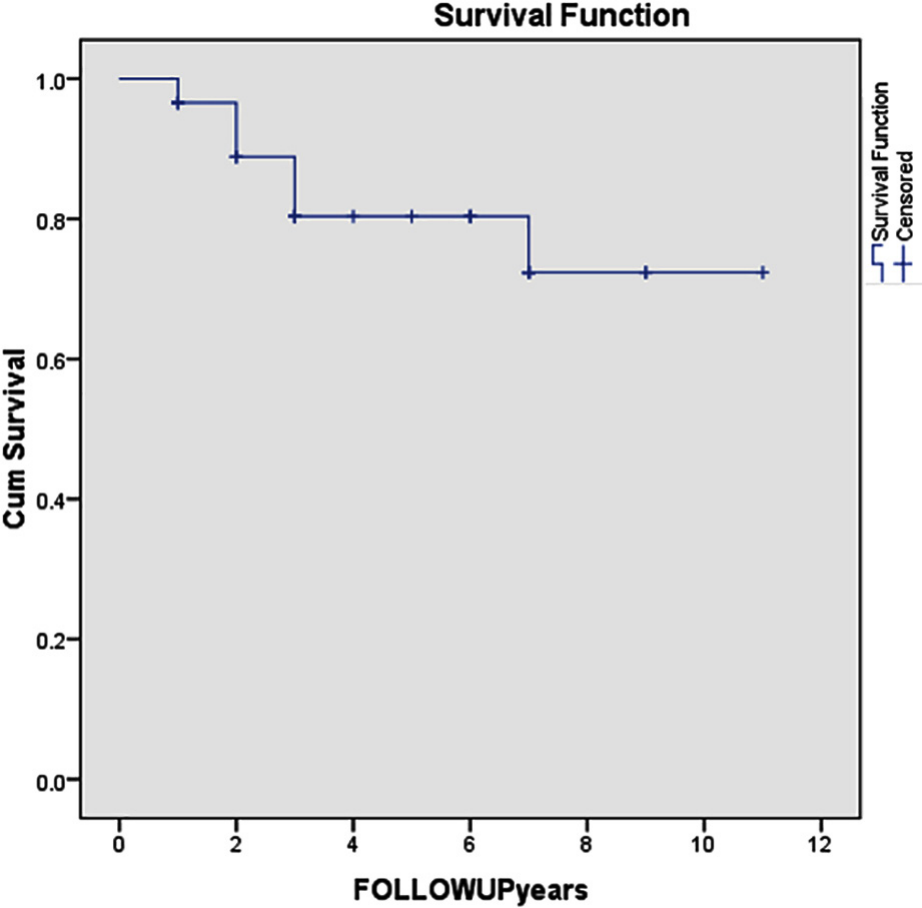
Kaplan–Meier survival function curve.

A total of 29 patients were included in the survival analysis. Over a maximum follow-up of 11 years, six deaths (20.7%) occurred, while 23 patients (79.3%) were censored. The Kaplan–Meier estimate of mean survival time was 8.96 years (95% CI: 7.54–10.39). The median survival time was not reached, as fewer than half of the cohort experienced the event. Our five-year survival rate was 73%. The Kaplan–Meier survival function curve can be observed from Fig.1 as provided in supplementary material.

## DISCUSSION

This study reviews our experience with blood vessel reconstruction as an integral part of surgery to remove musculoskeletal tumors involving major blood vessels located in the extremities. In this retrospective case series, surgical resection required vascular reconstruction with graft in 20 out of 29 patients while nine patients were dealt with primary vascular repair. Vascular involvement was not considered as a contraindication to sarcoma resection and whenever possible, venous reconstruction was performed. A multidisciplinary team was essential to our surgical approach. The preoperative team approach allowed for better surgical planning, excision of previously thought inoperable tumors based on outside hospital evaluations, more effective reconstruction, and improved outcomes. Preoperatively, patients met separately with the surgeon (surgical oncology or orthopedic surgeon), vascular surgeon, medical oncologist, and radiation oncologist. An integral part of this evaluation process was obtaining a computed tomography angiogram preoperatively and meeting with an angiogram and meeting with a vascular surgeon to plan the vascular reconstruction and resection.

Our study reported wound infection rate of 20.6% including both superficial and deep wounds. There was no statistically significant difference (p=1.0) in wound infection rates among the autogenous graft group, synthetic graft group, and primary repair group. Two patients received radiation therapy prior to resection, perhaps contributing to the development of infection and wound breakdown. Four patients with wound infection were managed conservatively with dressing and IV or oral antibiotics while two patients with deep infection required formal debridement in OR. Our findings are consistent with those of Adelani et al., who noted a 28.5% wound infection rate without any significant difference between synthetic and autogenous graft groups.^13^ Similarly, other retrospective studies have reported wound complication rates ranging from 22.2% to 29.4%, with complications occurring exclusively in patients with synthetic vascular grafts.^14^,^15^

There were two graft infections (6.89 %) in our study sample which were consistent with another cohort which reports incidence of graft infection as 14.2%.^13^ One patient with graft infection had to undergo revision of graft. However, the other patient with graft infection ended up in hip disarticulation due to complete vessel blockage and development of dry gangrene secondary to late presentation.

There were two early graft occlusions (6.89%). Both of the patients with graft thrombosis had undergone amputation due to limb ischemia and uncontrolled wound infection. Other studies have also reported a higher rate of early graft occlusions as 15.2%, 17.6%, 31.25% and 35.7%.^13^-^17^ Some authors report a significant association between graft occlusion and use of synthetic grafts than saphenous grafts^8^, while others report no significant association between graft occlusion and use of synthetic grafts.^13^ In our study none of the patients developed occlusion in synthetic grafts, all occlusions were observed in autologous grafts, which may be explained by the use of diseased vein or technical issues like kinking in the autologous conduit. Compared to the autogenous graft, prosthetic graft despite having higher risk of infection, are less prone to kinking or intimal disease process.

We assessed the functional status of our patients using the Toronto Extremity Salvage Scoring system.^12^ The mean Toronto Extremity Salvage Score was calculated to be 84.28 (range 38-98) and if we grade our functional score, only 21% had a poor outcome while 79% had good to excellent results. A case control study reported the post-operative functional Toronto Extremity Salvage Scores of 78.5 in patients with vascular reconstruction and 84.2 in control group which had no vascular procedure^10^, while another retrospective study reported the mean Toronto Extremity Salvage Score of 82.^2^ Some studies have also used Musculoskeletal Tumor Society Score (MSTS) scoring system for reporting functional outcomes and have reported a mean musculoskeletal tumor society score (MSTS) score of 70% and 83.3% in patients undergoing vascular reconstruction.^15^,^17^

In this study, primary patency was 89.5% at a mean follow up 55.2 months. There is a continued need for long-term surveillance with duplex ultrasonography and clinical examination. Early detection of graft stenosis with findings on duplex ultrasonography of elevated velocities or a drop in serial ankle-brachial indexes indicate a need for reintervention. Reintervention can be performed with catheter-based balloon angioplasty without the need for a large operation while maintaining patency. Studies have reported primary patency rates of 58.3% at median follow-up of 34 months^11^, 100% at mean follow-up of 34.8 months^2^, 78.6% at long term follow-up^17^, 88.9% at two years follow-up^14^ and 89% at 32 months.^16^ In our study, patency was declared with the use of palpable distal pulses or normal contrast opacification of graft in surveillance CT scans.

Limb salvage was achieved in 82.7% of our patients. Schwarzbach et al., Shah et al. and Adelani et al. has reported limb salvage rates of 94.1%, 92.2% and 92.8% respectively^11^,^13^,^17^, while others have reported 100% salvage rates.^2^,^14^ Above knee amputation was done in five patients while one patient underwent hip disarticulation. The primary reason for amputation was graft infection in one patient, graft thrombosis with dry gangrene in two patients, deep wound infection leading to osteomyelitis in one patient and one patient underwent an amputation outside of our hospital setting due to local recurrence along with distant metastasis.

There were no early deaths (within 30 days). Late mortality (>30 days) was 20.7% due to distant metastatic disease. The overall mean survival of our study was 8.6 years which Is comparable to median overall survival of 5.3 years reported in retrospective view.^18^ Survival probability declined most sharply within the first three years of follow-up, dropping from 96.6% after the first event to 80.4% after the fifth event. After the final death at seven years, the survival curve plateaued at approximately 72% (±10.4%), with no further events observed during the rest of the follow-up. The high proportion of censored observations indicates that long-term survival estimates beyond seven years should be interpreted with caution. Our five-year survival rate was 73%. This five-year survival rate is comparable to those reported in other studies, such as 77.8% and 80%.^14^,^17^ It is notably higher than the survival rates of 62% observed in another cohort.^16^ Local recurrences occurred in four patients (13.8%) at a mean follow up of 40.5 months. These numbers are comparable to those reported in literature. Song et al and Tsukushi et al report a recurrence rate of 21% and 20% respectively.^6^,^19^ Tumor recurrences were aggressively resected, which contributed to an excellent overall survival.

This study provides comprehensive documentation of patient demographics, tumor characteristics, and vascular repair techniques. Moreover, the study reports both intraoperative and postoperative complications along with functional outcomes, recurrence, metastasis, and tumor free interval, providing insights into patient outcomes. The findings can guide surgeons in optimizing perioperative care strategies to decrease complication rate, improving patient selection and management protocols for complex limb salvage procedures in resource-limited settings.

## CONCLUSION

Limb salvage surgery is a complex procedure, and it is only achievable if we work as a team, that is why it is important for such patients to be treated in a set up where multimodal approach can be applied. Post-operative complications such as wound infection, graft infection, or graft failure may occur; however, they are manageable and functional outcomes vary from patient to patient. In our study we observed overall good functional status in majority of patients. Amputation with prosthetic limb is however still a viable option, and should be kept as a last resort, especially at a tertiary care hospital like ours.

### Authors’ Contribution:

**NAS:** Conceptualization of the study, critical review

**JS:** Data analysis, editing, final review of the manuscript.

**BZ:** Data collection, manuscript writing

**MU:** Critical review.

All authors have approved the final version of manuscript and are accountable for the integrity of the study.
